# Sustained exposure to catecholamines affects cAMP/PKA compartmentalised signalling in adult rat ventricular myocytes

**DOI:** 10.1016/j.cellsig.2015.10.003

**Published:** 2016-07

**Authors:** Laura A. Fields, Andreas Koschinski, Manuela Zaccolo

**Affiliations:** a Institute of Neuroscience and Psychology, University of Glasgow, Glasgow, UK; b Department of Physiology, Anatomy and Genetics, University of Oxford, Oxford, UK

**Keywords:** CAMP, Phosphodiesterases, Cardiac hypertrophy, Protein kinase a, β-AR, β-adrenergic receptor, AKAP, A Kinase anchoring proteins, ANOVA, analysis of variance, ANP, atrial natriuretic peptide, ARVM, adult rat ventricular myocytes, cAMP, 3′, 5′ cyclic adenosine monophosphate, CFP, cyan fluorescent protein, D/D, dimerization docking, ECC, excitation-contraction coupling, EPAC, exchange proteins directly activated by cAMP, FRET, fluorescence resonance energy transfer, Frsk, Forskolin, GPCR, G protein-coupled receptors, IBMX, 3-isobutyl-1-methylxantine, ISO, isoproterenol, NE, norepinephrine, PCC, Pearson's correlation coefficient, PCR, polymerase chain reaction, PDE, phosphodiesterase, PKA, cAMP-dependent protein kinase, YFP, yellow fluorescent protein

## Abstract

In the heart compartmentalisation of cAMP/protein kinase A (PKA) signalling is necessary to achieve a specific functional outcome in response to different hormonal stimuli. Chronic exposure to catecholamines is known to be detrimental to the heart and disrupted compartmentalisation of cAMP signalling has been associated to heart disease. However, in most cases it remains unclear whether altered local cAMP signalling is an adaptive response, a consequence of the disease or whether it contributes to the pathogenetic process. We have previously demonstrated that isoforms of PKA expressed in cardiac myocytes, PKA-I and PKA-II, localise to different subcellular compartments and are selectively activated by spatially confined pools of cAMP, resulting in phosphorylation of distinct downstream targets. Here we investigate cAMP signalling in an *in vitro* model of hypertrophy in primary adult rat ventricular myocytes. By using a real time imaging approach and targeted reporters we find that that sustained exposure to catecholamines can directly affect cAMP/PKA compartmentalisation. This appears to involve a complex mechanism including both changes in the subcellular localisation of individual phosphodiesterase (PDE) isoforms as well as the relocalisation of PKA isoforms. As a result, the preferential coupling of PKA subsets with different PDEs is altered resulting in a significant difference in the level of cAMP the kinase is exposed to, with potential impact on phosphorylation of downstream targets.

## Highlights

• Sustained *in vitro* exposure of cardiac myocytes to catecholamines results in significant disruption of compartmentalised cAMP signalling.
• Altered compartmentalisation is due to relocation of both PKA isoforms and of phosphodiesterases within the myocyte.
• Relocalised PKA is exposed to different levels of cAMP.
• Altered compartmentalisation of cAMP/PKA signalling may be an early event that contributes to the development of cardiac remodeling.

## 1 Introduction

cAMP signalling mediates the catecholamine-dependent regulation of heart rate and contraction by modulating multiple aspects of the excitation contraction coupling (ECC) machinery [1]. The main effector of such regulation is protein kinase A (PKA), a tetrameric enzyme formed by two regulatory (R) and two catalytic (C) subunits. Ventricular myocytes express two PKA isoforms, PKA-I and PKA-II, which differ in their sensitivity to cAMP and in their subcellular localisation. On binding of β-ARs by catecholamines, adenylyl cyclases are activated to generate cAMP; cAMP in turn binds to the R subunits of PKA releasing active C subunits. The active enzyme phosphorylates several proteins involved in ECC including the L-type Ca^2 +^ channels, the ryanodine receptor, troponin I, myosin binding protein C and phospholamban, resulting in increased contractility and enhanced relaxation of ventricular myocytes [1]. This sequence of events is fundamental in the ability of the heart to finely adjust its output in response to continuous oscillations in oxygen demand by peripheral tissues and to react to stress situations with the ‘flight or fight’ response. However, persistent catecholamine stimulation of the heart is known to result in detrimental effects including cardiac remodelling and cardiac hypertrophy, leading ultimately to heart failure [2], [3], [4], [5].

In addition to regulation of contractility, cAMP mediates multiple other physiological functions within the cardiac myocyte, including gene transcription, with potentially negative long term effects [6]. A number of studies conducted over recent years support a model where spatial and temporal regulation of cAMP signals is paramount in maintaining normal function and where accuracy of signalling is achieved *via* compartmentalisation of cAMP and of the other molecular components of the pathways that are involved in cAMP signal propagation [7], [8]. This model is based on the notion that the raise in cAMP generated by hormonal stimulation is confined to distinct subcellular compartments, such that the cyclic nucleotide can only activate a limited subset of PKA enzymes, leading to phosphorylation of a selected number of substrates, thus achieving the appropriate functional outcome [9]. Critical to such compartmentalisation of signalling is the anchoring of PKA to specific subcellular sites [7] and the ability of phosphodiesterases (PDEs), the enzymes that degrade cAMP, to regulate locally the level of cAMP [10], [11].

Anchoring of PKA is achieved *via* binding of the R subunits to A kinase anchoring proteins (AKAPs), a large family of structurally unrelated proteins that have in common the ability to bind to PKA and thereby tether the enzyme to a specific location within the cell [12], bringing it in close proximity to a specific target. Anchoring of PKA to AKAPs is achieved *via* interaction of the N-terminal dimerization/docking (D/D) domain of the R subunit with an amphipathic helix of 14–18 residues within the AKAP [13]. The majority of AKAPs identified to date bind selectively to PKA-II [12], although dual-specific AKAPs as well as several PKA-I specific AKAPs have been reported [14], [15].

The role of PDEs in shaping local cAMP signals has long been recognised [16]. PDEs provide the only means of hydrolysing cAMP and can restrict the diffusion of cAMP thus preventing unspecific activation of individual PKA subsets. Multiple genes and splice variants for generating PDE isoforms targeted to distinct intracellular structures exist [17] and individual PDE isoforms are known to be under the control of a variety of different regulatory mechanisms. Thus, changing the amount and activation status of PDEs associated with a specific subcellular microdomain provides an important regulatory mechanism for local cAMP signals.

Given the high sophistication of the system underpinning compartmentalised cAMP signalling it is not surprising that alterations of its local control have been found to be associated to cardiac pathology. For example, a mutation affecting the ability of the AKAP Yotiao to interact with PKA leads to long QT syndrome [18] and alterations in PDE expression and activity have been found in several animal models of heart disease as well as in failing human hearts [19], [20]. In most cases however, it has not been possible to firmly establish whether the disrupted compartmentalisation of cAMP signalling is involved in the pathogenesis of the heart condition or whether it is a sequel of cardiac tissue remodelling that develops as a consequence of the disease. In this study we investigate whether sustained exposure of ventricular myocytes to high levels of catecholamines may directly affect cAMP compartmentalisation.

## 2 Material and methods

### 2.1 Reagents

Norepinephrine, cilostamide, rolipram, forskolin, were obtained from Sigma-Aldrich, BAY 60-7550 was from Cayman Chem. Phosphate-Buffered Saline (PBS), DMEM High Glucose, MEM199, Penicilline/Streptomycine (10,000 units of penicillin (base) and 10,000 μg of streptomycin (base)/ml) and Glutamine were purchased from Invitrogen.

### 2.2 Antibodies

Mouse α-actinin was purchased from Sigma (A7811), anti-PDE2A (PD2A-101AP) and anti-PDE3A (PD3A-101AP) were from FabGennix. Anti-PDE4B and anti-PDE4D were kindly provided by M Houslay. Anti-mouse IgG (A5278), anti-rabbit IgG (A8275) and anti-goat IgG (A8919) were from Sigma. Donkey anti-goat AlexaFluor® 488 (A11055), donkey anti-rabbit AlexaFluor® 488 (A21206) and goat anti-mouse AlexaFluor® 564 (A11055) were from Molecular Probes (Invitriogen).

### 2.3 ARVMs culture and adenoviral delivery

All animal procedures in this study were carried out according to the Home Office regulations regarding experiments with animals in the UK. Male Wistar rats (200 g–250 g) were sacrificed by cervical dislocation. Single cardiomyocytes were isolated as previously described [21], [22]. Briefly, hearts were removed, cannulated, perfused and digested with KREBS solution containing 0.66 mg/ml collagenase type I (Worthington BioChem) and 0.04 mg/ml protease type XIV (Sigma). Isolated cardiomyocytes were resuspended in MEM199 medium supplemented with 5 mM creatine (Sigma), 5 mM taurine (Sigma), 2 mM carnitine (Sigma), 1% Pen/Strep. Cells were seeded at 50,000 cells per 24 mm coverslips coated with laminin (Millipore) and incubated at 37 °C. After 2 h, cardiomyocytes were transduced with AdV5/CMV/RI_epac or AdV5/CMV/RII_epac (MOI 1000) for 24 h.

### 2.4 *In-vitro* hypertrophy protocol

For hypertrophy induction, ARVMs were treated with 1 μM norepinephrine (NE) in serum free medium. Cells were cultured for 24 h before imaging.

### 2.5 Generation of AdV5/CMV/RI_epac and AdV5/CMV/RII_epac

RI_epac and RII_epac constructs [9] were cloned into the pShuttle-CMV vector and transferred by homologous recombination into the pAdEasy-1 vector to generate AdV5/CMV/RI_epac and AdV5/CMV/RII_epac using AdEasy™ XL Adenoviral Vector System (Agilent Technologies — Stratagene Products), according to manufacturer's instructions.

### 2.6 Real-time PCR

Total RNA was extracted from cultured ARVM using TRIzol reagent (Invitrogen) which contained phenol and guanidine thiocyanate in a procedure based on the method of Chomczynski and Sacchi [23]. Reverse transcription of RNA samples was carried out using QuantiTect® Reverse Transcription Kit (Qiagen) according to manufacturer's instructions.

Gene-specific TaqMan probes and PCR primers sets were designed and purchased from Eurofins MWG operon. Real-time PCR was performed from reverse transcribed cDNA samples using the Platinum Quantitative PCR SuperMix-UDG with ROX (Invitrogen) following the manufacturer's instructions. qPCR reactions were conducted using the ABI Prism 7300 (Applied Biosystems) qPCR thermocycler and analysis software (95 °C for 2 min, followed by 40 cycles of 95 °C for 15 s, 57 °C for 15 s, 60 °C for 1 min). 18S rRNA was used as an internal control.

18S rRNA primers and TaqMan probe sequences;

Forward: 5′-CGCGGTTCTATTTTGTTGGT-3′,

Reverse: 5′-CGGTCCAAGAATTTCACCTC-3′,

TaqMan: 5′-FAM-TGAGGCCATGATTAAGAGGG-TAM-3′.

Atrial natriuretic peptide (ANP) primers and TaqMan probe sequences;

Forward: 5′-GGATTGGAGCCCAGAGCGGAC-3′,

Reverse: 5′-CGCAAGGGCTTGGGATCTTTTGC-3′,

TaqMan: 5′-FAM-AGGCTGCAACAGCTTCCGGT-TAM-3′.

α-actin primers and TaqMan probe sequences;

Forward: 5′- TCACCAAGCAGGAGTACGAC-3′,

Reverse: 5′- AGAGAGAGCGCGTACACAGA-3′,

TaqMan: 5′-FAM-ATGCTTCTAGGCGCACCCGC-TAM-3′.

Three biological replicates each of which included a minimum of three technical replicates were performed. The relative quantity of each transcript was determined using the comparative Ct method by interpolating the Ct values of the unknown samples to each standard curve [24]. Values were normalised with respect to 18S gene.

### 2.7 Cell size measurements

Single ARVM were chosen randomly and captured in bright field using an ORCA AG (model C4742-80-12AG) camera on the stage of an inverted epifluorescence microscope (Olympus IX81, equipped with an Olympus PlanApoN, 60X, NA 1.42 oil objective) and analysed. Cell size was determined by calculating the sectional area as length of the cell by width of the cell. One pixel corresponds to 0.116 × 0.116 μm.

### 2.8 FRET imaging

FRET imaging experiments were performed 24 h after ARVMs transduction with the AdV5/CMV/RI_epac or AdV5/CMV/RII_epac virus. During imaging cells were maintained at room temperature in a modified Ringer solution (NaCl 125 mM, KCl 5 mM, Na_3_PO_4_ 1 mM, MgSO_4_ 1 mM, Hepes 20 mM, Glucose 5.5 mM, CaCl_2_ 1 mM, pH 7.4), and imaged on an inverted microscope (Olympus IX81) using a PlanApoN, 60X, NA 1.42 oil immersion objective, 0.17/FN 26.5 (Olympus, UK).

The microscope was equipped with an ORCA-AG CCD camera (C4772-80- 12AG, Hamamatsu Photonics, UK) and a beam-splitter optical device (Dual-view simultaneous imaging system, DV2 mag biosystem, Photometrics, ET-04-EM). FRET filter settings used were: CFP excitation filter ET436/20 ×, dichroic mirror 455DCLP (Chroma Technology) in the microscope filter cube; dichroic mirror 505DCLP, YFP emission filter 545 nm, CFP emission filter 480 nm (Chroma Technology) in the beam splitter. Images were acquired using CELLΛ R software (Olympus) and processed using ImageJ. FRET changes were measured as changes in the background-subtracted 480/545 nm fluorescence emission intensity on excitation at 430 nm and expressed as either R/R_0_, where R is the ratio at time t and R_0_ is the ratio at time = 0 s, or ΔR/R_0_, where ΔR = R–R_0_. Values are expressed as the mean ± SEM.

### 2.9 Phosphodiesterase activity assay

Measurement of PDE activity was carried out using a radioactive cyclic AMP hydrolysis assay as previously described [25] and is a modification of a two-step procedure [26].

ARVM were homogenised in lysis Buffer (50 mM KCl, 50 mM Hepes, 1.94 mM MgCl_2_, 10 mM EGTA, 1 nM DTT) and protein concentration quantified by Bradford assay. 20–25 μg of purified protein was used per sample. Samples were assayed in a reaction mixture containing 40 mM Tris–HCl (pH 8.0), 1 mM MgCl_2_, 1.4 mM β-mercaptoethanol, 2 μM cAMP and 3 μCi/ml of 8-[3 H]cAMP for 10 min at 30 °C. The reaction was terminated by heat inactivation in a boiling water bath for 2 min. The PDE reaction product 5′-AMP was then hydrolysed by with 50 μg of *Crotalus atrox* snake venom for 10 min at 30 °C, and then separated by anion exchange chromatography using a Dowex/ethanol mix. Samples were quantified by scintillation counting. Individual PDE activities were defined as the fraction of cAMP-PDE activity inhibited by 50 nM Bay 60–7550, 10 μM cilostamide, and 10 μM rolipram. DMSO and non-selective PDE inhibitor IBMX (10 μM) were used as controls. Specific PDE activity was determined as pmol cAMP hydrolysed/min/mg protein.

### 2.10 Immunostaining and confocal imaging

ARVM were transduced with adenovirus carrying RI_epac or RII_epac as described above and cultured for 24 h. For PDE localisation experiments, non-transduced myocytes were used. Cells were fixed with ice cold methanol, permeabilised in PBS containing 0.1% Triton®X-100 and incubated for 30 min with blocking buffer (PBS containing 1% BSA). Primary antibodies were diluted in blocking buffer and incubated overnight at 4 °C. For each coverslip treated with primary antibody, an IgG control was prepared.

Confocal images were acquired using a 63 × Zeiss oil immersion objective on a Zeiss Pascal LSM510 laser-scanning confocal microscope (Carl Zeiss). An argon laser was used to excite AlexaFluor® 488-conjugated donkey anti-rabbit IgG or Alexa Fluor® 488-conjugated donkey anti-goat IgG secondary antibodies. Helium/neon lasers were used to excite goat anti-mouse AlexaFluor® 568. Zeiss Pascal software was used to collect images. Pearson's correlation coefficient was calculated using ImageJ software with JACoP plugin.

### 2.11 Statistical analysis

Data are expressed as mean ± SEM. Differences between multiple groups were compared by analysis of variance (ANOVA) followed by multiple comparisons post-test, as indicated. Two-group analysis was performed by t-test. Number of replicates is indicated in the figure legends. *p ≤ 0.05; **p ≤ 0.01; ***p ≤ 0.001; ns = not significant.

## 3 Results

**Fig. 1:**
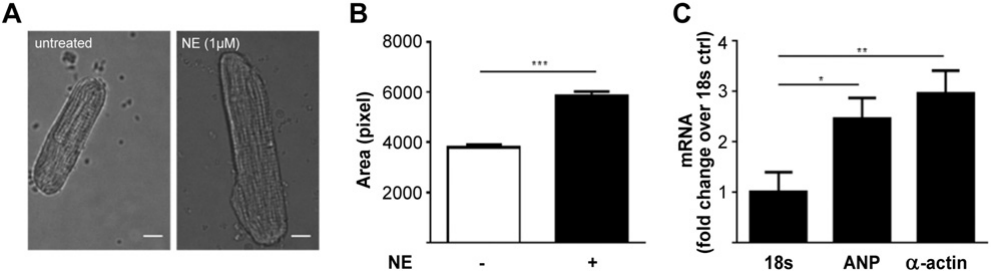
Adult rat ventricular myocytes treated with norepinephine (NE) for 24 h develop hypertrophy. (A) Representative images of adult rat ventricular myocytes (ARVM) untreated and after 24 h treatment with NE (1 μM). Scale bar is 10 μm. (B) Longitudinal section area of ARVM calculated for untreated control or NE (1 μM) treated cells. n ≥ 90. Pixel size = 0.116 × 0.116 μm. (C) mRNA levels for ANP and α-actin in NE (1 μM) treated ARVM. Ribosomal 18 s subunit mRNA was used as control. n ≥ 7. Data expressed as mean ± SEM. Two tailed paired t-test performed for panel B. One way ANOVA with Dunnett's post-test performed for panel C.

To assess whether compartmentalised cAMP signalling may be altered by sustained exposure to catecholamines we treated adult rat ventricular myocytes (ARVM) with 1 μM norepinephrine (NE) for 24 h, a well-established protocol to induce cardiac myocyte hypertrophy *in vitro* [27], [28], [29]. As expected, after treatment the cells displayed increased size (Fig. 1A, B) and reactivation of the foetal gene programme, as illustrated by increased mRNA levels for atrial natriuretic peptide (ANP) and skeletal α-actin (Fig. 1C), confirming that this protocol induces a modification of the cellular phenotype that recapitulates a number of changes observed in hypertrophic hearts *in vivo* [30]*.*

### 3.1 Hypertrophic cardiac myocytes show altered distribution of PKA-I and PKA-II binding sites

**Fig. 2:**
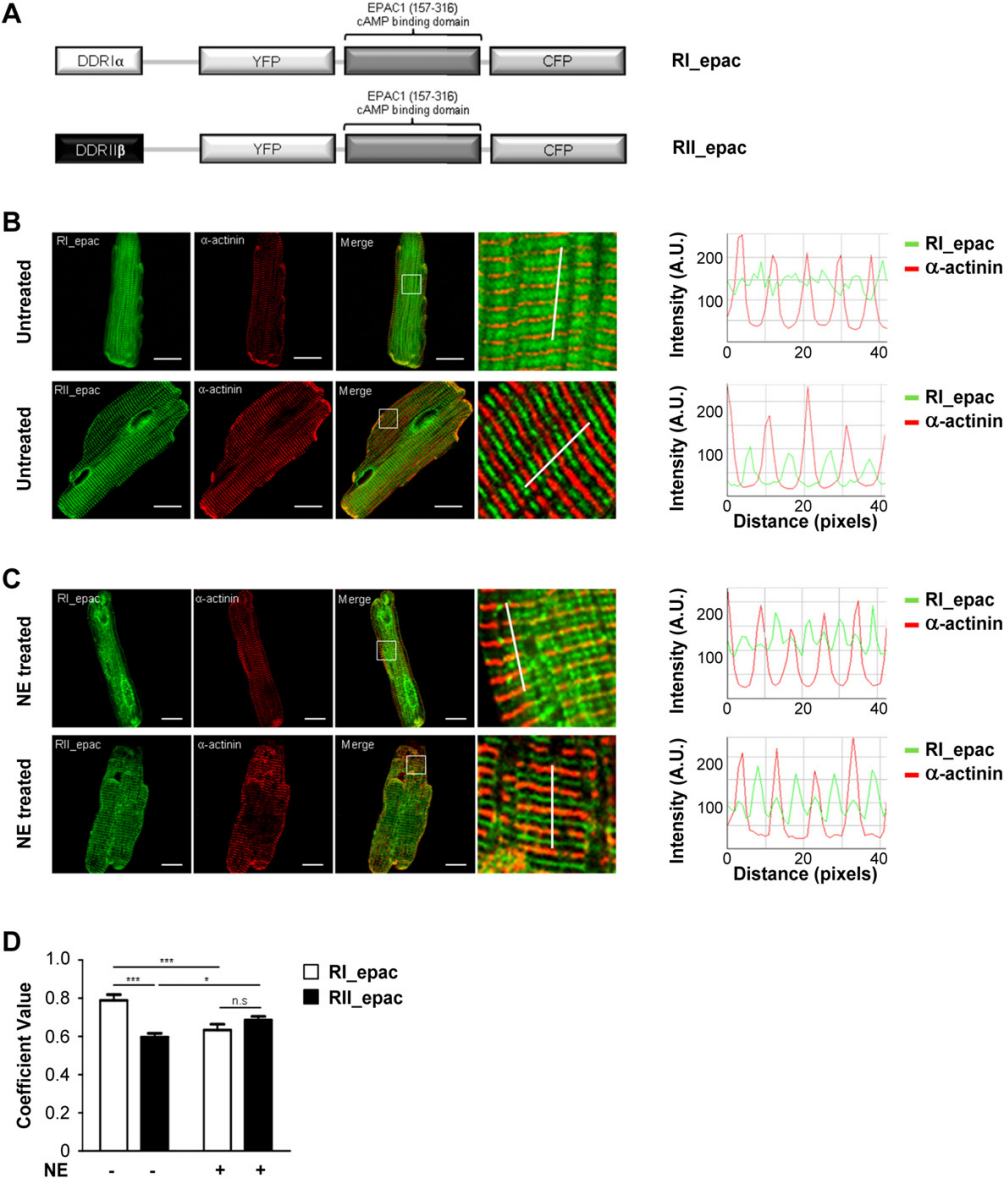
Localisation of RI_epac and RII_epac reporters. (A) Schematic representation of the FRET reporters RI_epac and RII_epac. The dimerisation/docking domains from PKA-RIα and PKA-RIIβ, which mediates anchoring of the sensors to specific AKAPs, are shown in white and black, respectively. (B) Left panels: confocal images illustrating the localisation of RI_epac or RII_epac (in green) and the reference marker sarcomeric α-actinin (in red) in ARVM expressing the sensor in culture for 24 h without any further treatment. Right panels: line intensity profiles for RI_epac and RII_epac (in green) and α-sarcomeric actinin (in red). The position of the line where the intensity values were calculated is shown in the corresponding magnified panels on the left. (C) Samples are as in A except that cells were treated for 24 h with NE (1 μM). (D) Pearson's correlation coefficient calculated for RI_epac or RII_epac and sarcomeric α-actinin in untreated and NE-treated ARVM. n ≥ 7. Data expressed as mean ± SEM. Two-way ANOVA with Bonferroni multiple comparisons tests was performed. Scale bars are 10 μm.

To assess whether sustained exposure to catecholamines may have an effect on the localisation of PKA isoforms we used the RI_epac and RII_epac FRET-based cAMP sensors [9]. As illustrated in Fig. 2A these sensors include the D/D domain from PKA-I and PKA-II, respectively, fused at the amino-terminus of the Epac1-camps cAMP FRET reporter [31]. The presence of the isoform-specific D/D domain confers to the sensor the ability to selectively bind to the intracellular sites where PKA-I and PKA-II normally bind within cardiac myocytes [9], [21]. ARVM expressing RI_epac or RII_epac and immuno-labelled with an antibody to α-actinin, a protein localised at the sarcomeric Z line, were analysed by confocal microscopy. The specific localisation of the sensors was assessed by estimating the degree of their relocation relative to α-actinin by calculating Pearson's correlation coefficient (PCC) values. As shown in Fig. 2B and 2C, RI_epac and RII_epac show a clearly distinct localisation in untreated cells (PCC values of 0.79 ± 0.03 for RI_epac, n = 15, and 0.60 ± 0.02 for RII_epac, n = 13, p = 0.0001). Sustained treatment with NE significantly altered the localisation of both sensors as evident from the fluorescence intensity line scan analysis (Fig. 2C) and as confirmed by PCC values (Fig. 2D; p = 0.0003 and p = 0.0426 for RI_epac and RII_epac, respectively). The relocalisation of PKA-I and PKA-II sites in hypertrophic cells completely abolished the difference between the two sensors present in untreated cells (RI_epac: 0.63 ± 0.03, 9; RII_epac: 0.69 ± 0.02, n = 9, p = 0.34).

### 3.2 The amplitude and compartmentalisation of cAMP signals is affected in hypertrophic cardiac myocytes

**Fig. 3:**
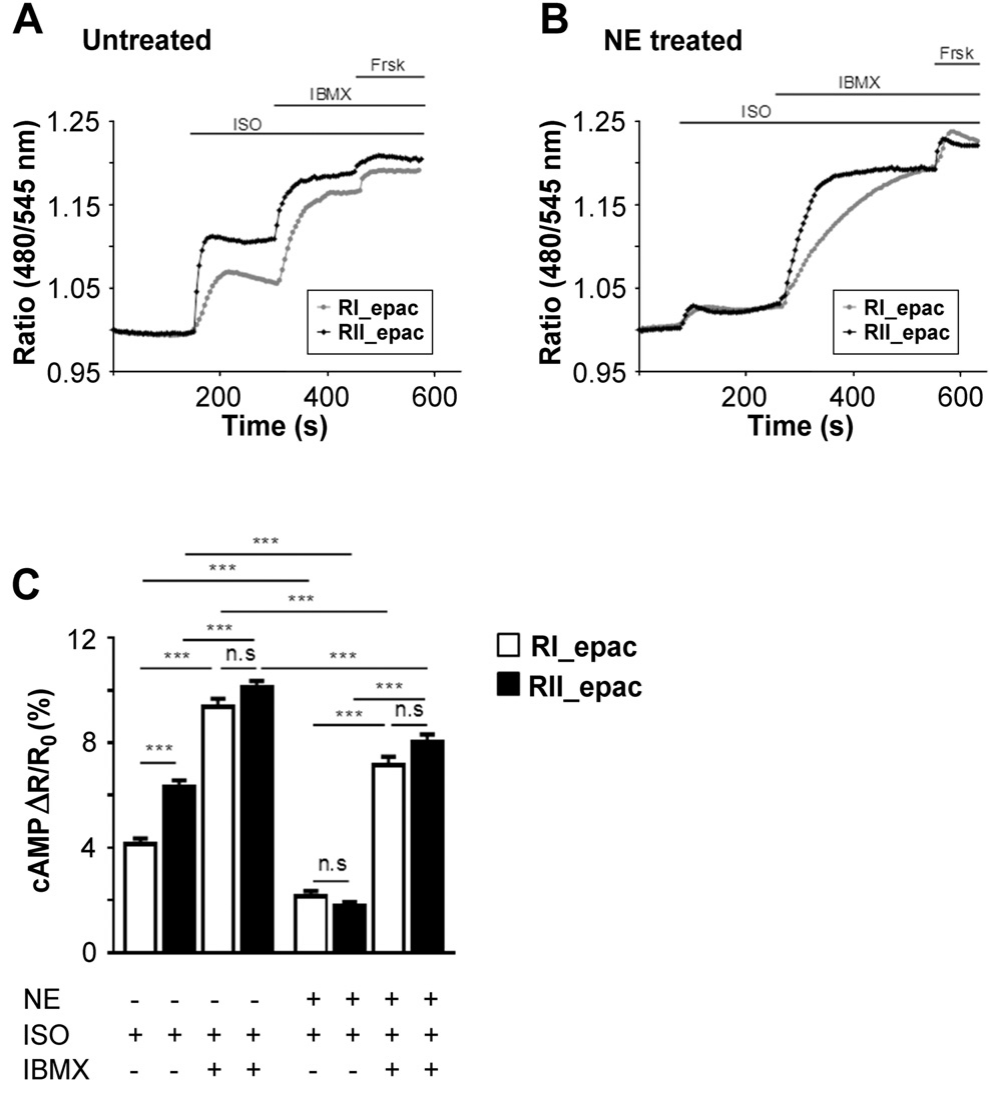
Effect on local cAMP signals of sustained exposure of ARVM to catecholamines. Representative kinetics of cAMP changes detected in the PKA-I and PKA-II domains in response to ISO (100 nM), IBMX (10 μM) and forskolin (25 μM) in RI_epac or RII_epac expressing (A) untreated ARVM or (B) ARVM treated for 24 h with NE (1 μM) to induce hypertrophy. At the end of the experiment 25 μM forskolin was applied to achieve saturation of the sensor. Ratio values are calculated as R/R_0_. (C) Summary of data measured as shown in A) and B) and expressed as increment of FRET signal over basal. n ≥ 15. Data are mean ± SEM. Two-way ANOVA with Tukey's multiple comparisons tests were performed.

To assess whether sustained exposure to catecholamines may affect the level of cAMP that is sensed by individual PKA isoforms in response to β-AR stimulation, we performed FRET-based imaging of ARVM expressing RI_epac or RII_epac. Untreated cells were challenged with isoproterenol (ISO, 100 nM) and the amplitude of FRET change was monitored in the PKA-RI and PKA-RII subcellular compartments. The sensor targeted to the PKA-II compartment detected a significantly larger increase in cAMP as compared to the sensor targeted to the PKA-RI compartment (ΔR/R_0_ = 4.13 ± 0.22%, n = 26 for RI_epac and ΔR/R_0_ = 6.20 ± 0.22%, n = 31 for RII_epac: p < 0.0001) (Fig. 3A, C). When, in the presence of ISO, the PDEs where inhibited with the non-selective inhibitor IBMX, a larger FRET change was detected in the PKA-I compartment as compared to the PKA-II compartment resulting in abrogation of the differences in cAMP levels in the two compartments (RI_epac ΔR/R_0_ = 9.38 ± 0.31%, n = 26; RII_epac ΔR/R_0_ = 10.11 ± 0.25%, n = 27; p = 0.403) (Fig. 2A, C). These data indicate that in non-hypertrophic cells ISO stimulation generates a compartmentalised cAMP response that depends on a greater PDEs activity associated with the PKA-RI compartment compared to the PKA-RII. In striking contrast, the cAMP response to ISO in hypertrophied cells was largely blunted compared to controls and identical in the two compartments (Fig. 3B, C) (ΔR/R0 = 2.137 ± 0.215% for RI_epac, n = 28 and ΔR/R0 = 1.774 ± 0.146% for RII_epac, n = 34; p = 0.9501). Non-selective PDE inhibition in hypertrophic myocytes resulted in a comparable FRET change in the two compartments (ΔR/R0 = 7.162 ± 0.316% for RI_epac, n = 26; ΔR/R0 = 8.023 ± 0.301% for RII_epac, n = 15, p = 0.4139). These results together indicate that sustained exposure to catecholamines results in largely reduced cAMP levels in response to β-ARs stimulation, an effect that is particularly prominent in the PKA-II domain, and the compartmentalisation of the cAMP signal is abolished.

### 3.3 Sustained exposure to catecholamines does not affect the overall activity of individual PDEs

**Fig. 4:**
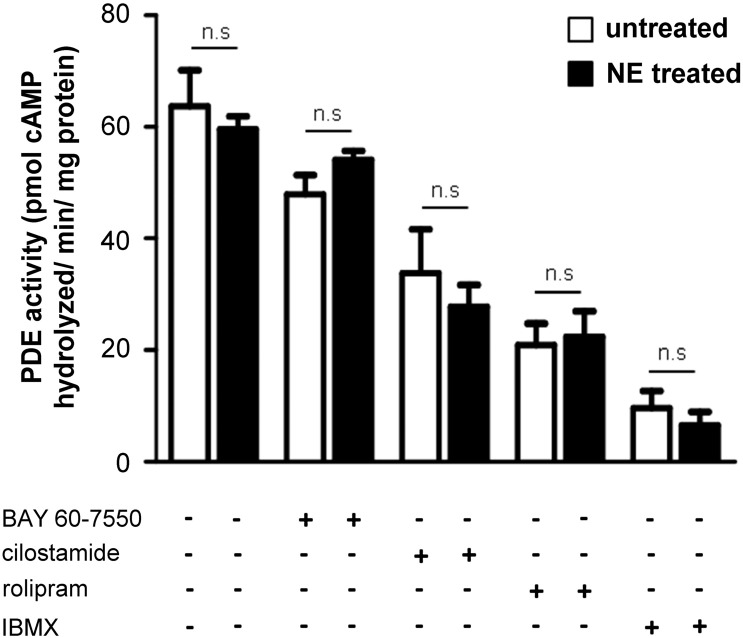
Overall PDE activity in untreated and hypertrophic myocytes. PDE activity was measured by radio-enzymatic assay using 2 μM cAMP as substrate. Cell lysates from untreated and NE-hypertrophied ARVM were assessed in the presence of PDE inhibitors Bay 60–7550 (50 nM), cilostamide (10 μM), rolipram (10 μM) or IBMX (10 μM). n ≥ 5. Data represent mean ± SEM. Two-way ANOVA with Bonferroni post-tests were performed.

**Fig. 5:**
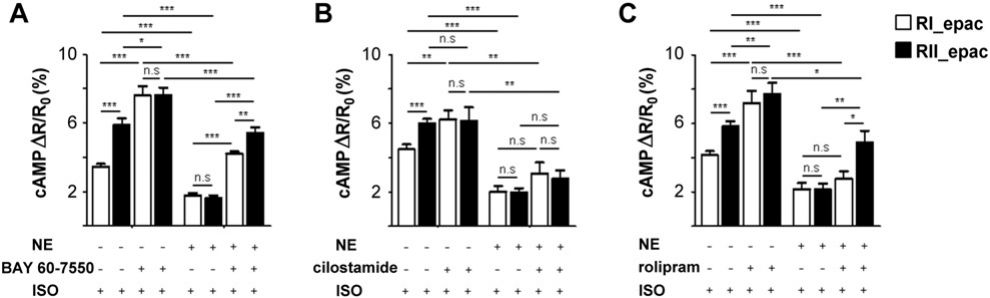
Contribution of individual PDEs to the local regulation of cAMP. FRET measurements of cAMP levels generated in response to ISO (100 nM) by untreated and NE-treated ARVMs expressing either RI_epac or RII_epac and pre-incubated for 10 min with (A) Bay 60–7550 (50 nM), n ≥ 7; (B) cilostamide (10 μM), n ≥ 8; or (C) rolipram (10 μM), n ≥ 7. Data are expressed as mean ± SEM. Two-way ANOVA with Tukey's multiple comparisons tests were performed.

The above data indicate that sustained treatment of ARVM with catecholamines affects compartmentalisation of cAMP signals in the PKA-I and PKA-II domains. This may be due to the observed relocation of PKA-I and PKA-II induced by the treatment, resulting in coupling of the PKA isoforms with a different array of local PDEs. However, a change in the overall activity of individual PDEs, resulting from increased level of enzyme expression, or an effect on the local regulation of individual PDEs could also be a contributing factor. To assess whether sustained exposure to catecholamines affects the overall activity of PDEs, a PDE activity assays was performed on cell lysates of hypertrophic and control ARVM. Lysates were treated with PDE inhibitors and the amount of cAMP hydrolysis was calculated. As expected, non-selective PDE inhibition with IBMX resulted in a large decrease in cAMP hydrolysing activity (Fig. 5). Application of selective PDE inhibitors showed decreasing overall hydrolysing activity for PDE4, PDE3 and PDE2 (Fig. 4). However, no difference was detected between untreated and hypertrophic myocytes for all inhibitors (Fig. 4).

### 3.4 Hypertrophic myocytes show altered local PDE activity

To assess whether any specific PDEs may be primarily responsible for the altered compartmentalisation observed, untreated or NE-hypertrophied ARVM expressing either RI_epac or RII_epac were pre-incubated with a selective PDE inhibitor before challenge with 100 nM ISO. As shown in Fig. 5A selective inhibition of PDE2 with Bay 60 7550 (50 nM) increased the rise in cAMP in the PKA-I and PKA-II compartments in both untreated and hypertrophic cells. Selective inhibition of PDE3 with cilostamide (10 μM) resulted in a cAMP increase exclusively in the PKA-I domain of untreated cells whereas it appeared to have no detectable effect in hypertrophic myocytes (Fig. 5B). Pre-treatment of control ARVM with the PDE4 selective inhibitor rolipram (10 μM) showed an effect in both domains (Fig. 5C) whereas in hypertrophic myocytes the effect of PDE4 inhibition was detectable exclusively in the PKA-II compartment (Fig. 5C). These findings suggest that sustained exposure to catecholamines results in loss of PDE3 and PDE4 activity in the PKA-RI compartment and enhanced PDE4 activity in the PKA-RII compartment in the hypertrophic ARVM.

**Fig. 6:**
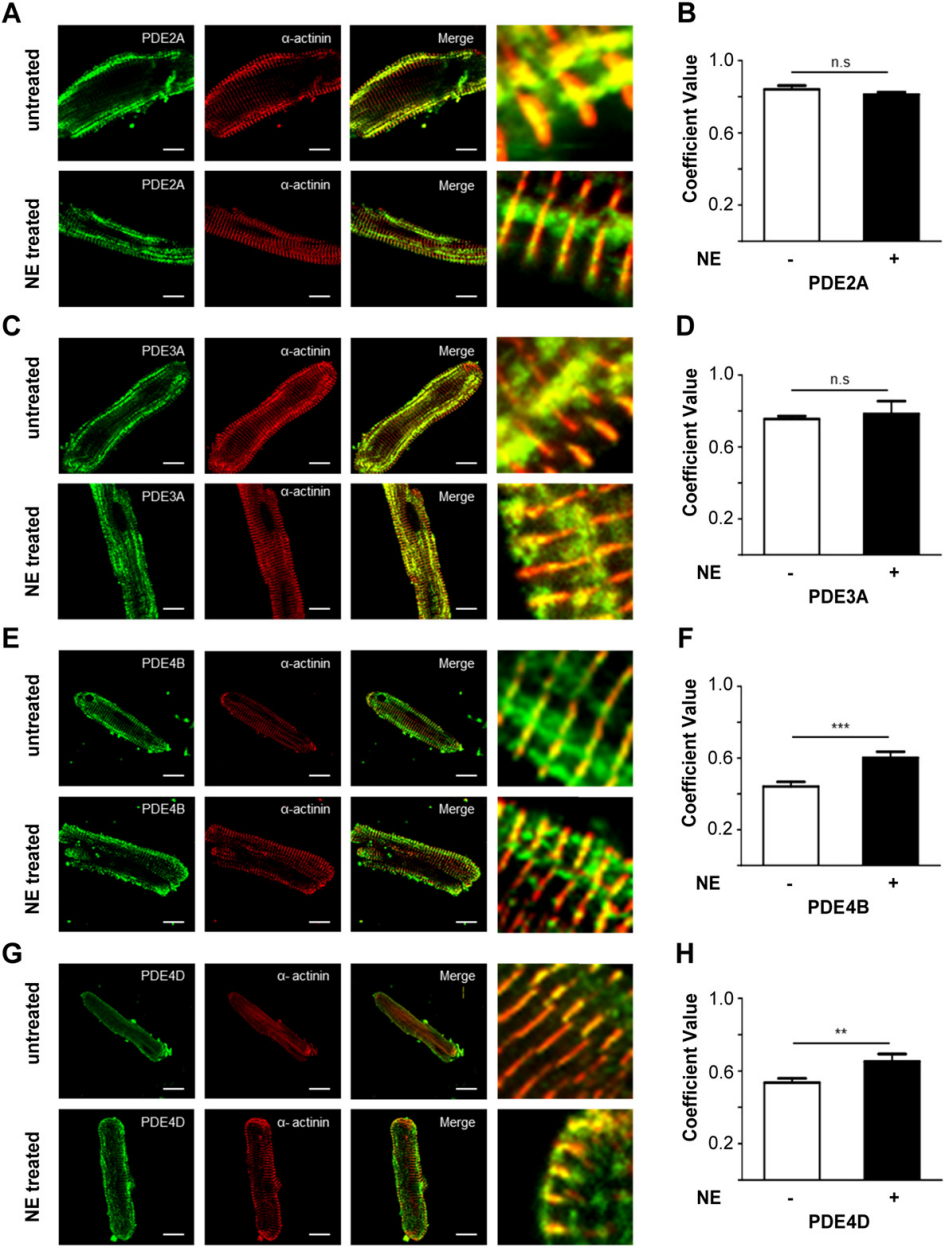
Localisation of individual PDE families in normal and hypertrophic myocytes. Confocal images of untreated (upper row) and NE-treated (lower row) ARVMs immunostained with anti-α-actinin (red) and (in green) antibodies to PDE2A (A), PDE3A (C), PDE4B (E) and PDE4D (G). The corresponding PCC's are shown in panels B, D, F and H, respectively. Images were acquired at the confocal microscope. Data expressed as mean ± SEM. Two tailed; unpaired t-tests were performed. For all experiments n ≥ 3.

To assess whether sustained exposure to catecholamine may affect the localisation of individual PDEs we performed immunofluorescence and confocal microscopy analysis of untreated and NE-treated ARVM decorated with anti-PDE (PDE2A, PDE3A, PDE4B or PDE4D) and anti-α-actinin antibodies and calculated the corresponding PCC values. As shown in Fig. 6, our analysis shows no difference between control and hypertrophic cells in the localisation for PDE2 and PDE3. Interestingly, the localisation of PDE4B and PDE4D appeared to be altered in hypertrophic myocytes where a higher correlation coefficient between PDE and α-actinin was detected, suggesting that sustained exposure to catecholamine may result in relocalisation of PDE4 isoforms to the Z line (Fig. 6F, PDE4B control cells: 0.44 ± 0.03, n = 20; PDE4B hypertrophic cells: 0.60 ± 0.03, n = 12, p = 0.0006; Fig. 6H, PDE4D control cells: 0.54 ± 0.02, n = 12; PDE4D hypertrophic cells: 0.65 ± 0.04, n = 7, p = 003).

## 4 Discussion

Enhanced activity of the sympathetic nervous system and the increased release of NE from the sympathetic nerve endings within the myocardium has long been recognised to be associated with a number of pathophysiological conditions leading to cardiac hypertrophy and heart failure [32]. In this study we used a simplified *in vitro* model of cardiac myocyte hypertrophy to explore whether sustained exposure to NE directly affects the activity of PDEs and/or the localisation of PKA isoforms with effects on compartmentalised cAMP signalling. For our ivestigation we used targeted cAMP sensors that allow monitoring of the cAMP level at subcellular sites were PKA-I and PKA-II are localised within ARVMs. We found that, in agreement with previous data generated in neonatal rat ventricular myocytes [9], in ARVM the two PKA isoforms are exposed to significantly different levels of cAMP in response to ISO stimulation and that PKA-II is the isoform that is exposed to the strongest cAMP signal. This confirms a high degree of compartmentalisation of cAMP in cardiac myocytes and the preferential engagement of PKA-II isoforms dowstream of β-AR activation, indicating that this isoform is the main effector of the cAMP-dependent regulation of ECC. In striking constrast, sustained exposure of ARVM to NE results in a dramatic reduction in the cAMP response to ISO and the difference in cAMP levels between the PKA-I and PKA-II domains is abolished. This appeares to be the consequence of two mechanisms: *i*) relocalisation of PKA and *ii*) a change in the coupling of the relocalised PKA isoforms with PDEs. The fact that the reduction in the strength of the cAMP signal predominantly affects the PKA-II domain is particularly relevant as this isoforms requires a higher concetration of cAMP to be activated compared to the PKA-I isoform [33]. Thus, it appears that sustained exposure to catecholamines abrogates the possibility to differentially activate the two PKA isoforms and that particularly the activation of PKA-II is compromised, with potential impact on the regulation of ECC.

Several studies have shown altered expression and/or activity of PDEs in cardiac hypertrophy and heart failure in a number of animal models as well as in human failing hearts. In general the data reported so far indicate a decreased activity of PDE3 and PDE4 [19], [34] and increased activity of PDE2 [22], [35], [36], although the exact PDE makeup of cardiac myocytes appears to vary depending on the stage of the disease [20]. Given the complexity of the *in vivo* processes leading to cardiac remodelling and heart failure, published studies have not been able to establish whether the alterd activity of PDEs observed is simply a bystander effect of the pathological remodelling, whether it represents an adaptive process or whether it contributes to the development of the disease (but see [22]). Our analysis suggests that the reduced cAMP response observed upon sustained exposure to catecholamines is due not only to the expected downregulation of β-ARs [37], but it is also the consequence of a redistribution of the PKA enzyme that brings PKA-II under a tighter control by PDEs. Indeed, we show a relocation of PDE4B and PDE4D enzymes to sites where also PKA-II appears to relocalise. Interestingly, relocalisation of PKA isoforms has been reported in human failing hearts [38].

In summary, our results indicate that sustained β-AR stimulation significantly affects cAMP compartmentalisation, it leads to redistribution of PKA isoforms and it abrogates the potential to differentially activate them. This may result in substantially reduced activation of PKA, and particularly of PKA-II, with potential consequences on the regulation of myocyte contractility. Our present findings suggest that the relocalisation of PKA isoforms and disruption of PDE-dependent compartmentalisation of cAMP signalling may be an early event in conditions of sustained adrenergic drive. The observed changes may significantly contribute to the reduced adrenergic reserve of the heart and therefore contribute to the pathogenetic process.
